# Silicone rubber membrane devices permit islet culture at high density without adverse effects

**DOI:** 10.3389/fbioe.2024.1401608

**Published:** 2024-07-11

**Authors:** Efstathios S. Avgoustiniatos, Kate R. Mueller, William E. Scott III, Jennifer P. Kitzmann, Thomas M. Suszynski, Brian E. Perrault, Eric J. Falde, A. N. Balamurugan, Bernhard J. Hering, Charles W. Putnam, Klearchos K. Papas

**Affiliations:** ^1^ Department of Surgery, Schulze Diabetes Institute, University of Minnesota, Minneapolis, MN, United States; ^2^ Department of Surgery, Institute for Cellular Transplantation, University of Arizona, Tucson, AZ, United States

**Keywords:** high-density islet culture, islet oxygenation, islet viability, islet recovery, G-Rex, silicone rubber membrane, gas-porous membrane, oxygenation model

## Abstract

**Introduction:**

Conventional culture conditions, such as in T-flasks, require that oxygen diffuse through the medium to reach the islets; in turn, islet surface area density is limited by oxygen availability. To culture a typical clinical islet preparation may require more than 20 T-175 flasks at the standard surface area density of 200 IE/cm^2^. To circumvent this logistical constraint, we tested islets cultured on top of silicon gas-permeable (GP) membranes which place islets in close proximity to ambient oxygen.

**Methods:**

Oxygenation of individual islets under three culture conditions, standard low-density, *non-*GP high density, and GP high density, were first modeled with finite element simulations. Porcine islets from 30 preparations were cultured for 2 days in devices with GP membrane bottoms or in paired cultures under conventional conditions. Islets were seeded at high density (HD, ∼4000 IE/cm^2^, as measured by DNA) in both GP and *non-*GP devices.

**Results:**

In simulations, individual islets under standard culture conditions and high density cultures on GP membranes were both well oxygenated whereas *non-*GP high density cultured islets were anoxic. Similarly, compared to the *non*-GP paired controls, islet viability and recovery were significantly increased in HD GP cultures. The diabetes reversal rate in nude diabetic mice was similar for HD GP devices and standard cultures but was minimal with *non*-GP HD cultures.

**Discussion:**

Culturing islets in GP devices allows for a 20-fold increase of islet surface area density, greatly simplifying the culture process while maintaining islet viability and metabolism.

## 1 Introduction

Following the promulgation of the Edmonton protocol (Shapiro et al., 2000), islet allotransplantation emerged as a promising treatment option for selected patients with type 1 diabetes (Hering et al., 2005; Shapiro et al., 2006; Bellin et al., 2008; Fiorina et al., 2008; Hogan et al., 2008). Alternative sources of beta cells, including stem cells, recently summarized in Mourad and Gianello (2017), or porcine islets for xenotransplantation (Mourad and Gianello, 2017), are also being pursued to increase the supply of islets. Culture of islets for research, pre-clinical animal studies, or clinical transplantation has become standard practice; it allows time for initiation of immunosuppression, potentially reduces immunogenicity by clearing endothelial cells and debris (Nason et al., 1988), and relaxes the logistical constraints of shipping. Current best practice for islet culture in conventional, *non-*gas permeable surfaces (e.g., T-flasks) limits islet equivalent (IE) seeding to 200 IE/cm^2^ surface area or 1,000 IE/mL volume density (Protocols, 2015). This constraint is imposed by the rate of diffusion of gases through the medium; the time required for oxygen to diffuse from the surface of the medium to the islets residing on the bottom of the flask increases with the depth of the medium, which is therefore typically limited to 0.2 cm.

Low density seeding of islet cultures attempts to strike a balance between the availability of oxygen in the medium and the logistical and economic demands of inoculating and maintaining large numbers of flasks. A typical porcine or human islet preparation may yield ≥600,000 IE; when plated at low surface density (i.e., 200 IE/cm^2^), more than 20 T-175 flasks are required. Seeding even a single islet preparation into a large number of flasks is labor-intensive, increases the risk of contamination, and imposes additional logistical and cost burdens on large-scale clinical islet manufacture. We reasoned that if the available oxygen content were substantially increased by culturing islets directly on the surface of a thin, liquid-impermeable, gas-permeable (GP) silicone rubber membrane (SRM), reducing the distance that ambient oxygen must diffuse to reach the islets (Yeager and Roy, 2017), the seeding density of islets could be substantially increased, thereby reducing the number of flasks required.

By applying the one-dimensional diffusion-reaction equation in planar geometry (Avgoustiniatos and Colton, 1997; Avgoustiniatos et al., 1997), we calculated that an entire human or porcine islet preparation of high purity would be fully oxygenated if cultured in one to two large (∼100 cm^2^) GP devices. After confirming this result with the more powerful finite-element model, we tested the concept by individually culturing three human islet preparations (230,000–430,000 IE each) in single large devices with no loss of viability compared to conventional, low-density cultures (Papas et al., 2005).

Here we characterize 2-day cultures from 30 porcine islet preparations seeded in conventional devices at low surface density as controls, and at high surface densities in GP and *non-*GP devices. Even at high density, islet cultures in GP devices had substantially improved viability and, importantly, increased metabolic activity.

## 2 Materials and methods

### 2.1 Animal ethics

All animal research was performed with the approval of and in accordance with guidelines of the University of Minnesota and the University of Arizona Institutional Animal Care and Use Committees (IACUC).

### 2.2 Modeling of oxygen profiles

Steady-state oxygen profiles were estimated using the COMSOL Multiphysics finite-element simulation package (COMSOL Inc., Burlington, MA). Details of the oxygen profiling model are provided in the Supplementary Material, Part 1 , Modeling of Oxygen Profiles. The silicone rubber membrane (SRM) thickness was assigned a value of 188 μm based on the average thickness of individual membranes from a single batch, mean 188 (SD 3) μm (Avgoustiniatos et al., 2008). Specific parameters are included in the relevant Results Sections 3.1.1, 3.1.2. Additional parameters used in the modeling are described elsewhere (Avgoustiniatos and Colton, 1997; Papas et al., 2005; Avgoustiniatos et al., 2007; Avgoustiniatos et al., 2008).

### 2.3 Preparation of porcine islets

Isolated porcine islets were prepared in accordance with previously described methods (Brandhorst et al., 1999; Kirchhof et al., 2004; Smith et al., 2018).

### 2.4 Two-day culture conditions

#### 2.4.1 Standard culture

Standard culture was carried out in untreated T-flasks (Sarstedt, Newton, NC), at ≤200 islet equivalents (IE)/cm^2^ (1,000 IE/mL), at 37°C, in air (no additional CO_2_). The base culture medium was custom-formulated Medium E199 with HEPES (Mediatech, Herndon, VA). Its supplementation was consistent for each experiment but changed somewhat over the multi-year course of the study. Typically, it was supplemented with sodium pyruvate, zinc sulfate, ITS Premix, ciprofloxacin, nicotinamide, and n-acetyl-L-cysteine and, within 24 h of use, with L-glutamine, heparin, and 10% heat-inactivated porcine serum (Invitrogen, Carlsbad, CA).

#### 2.4.2 High-density culture in *non-*GP devices

High-density culture in *non-*GP devices differed from standard culture in two ways: a) the surface density (IE/cm^2^) was greater; and b) in some instances instead of T-flasks, custom-made devices of smaller culture surface area (2.5, 5, 10, or 18 cm^2^) were used to reduce the number of islets needed to achieve high surface density. Although the latter devices had SRM bases, access to ambient air of the SRM base was blocked by sealing a *non-*GP membrane to the bottom. Cultures survived at equivalent rates in such devices as with T-flasks. In all cases, volume density was kept at 1,000 IE/mL, which is standard practice for a 2-day culture. This resulted in greater depths of medium for higher surface densities, further exacerbating the limitations imposed by oxygen diffusion.

#### 2.4.3 Culture in GP devices

**FIGURE 1:**
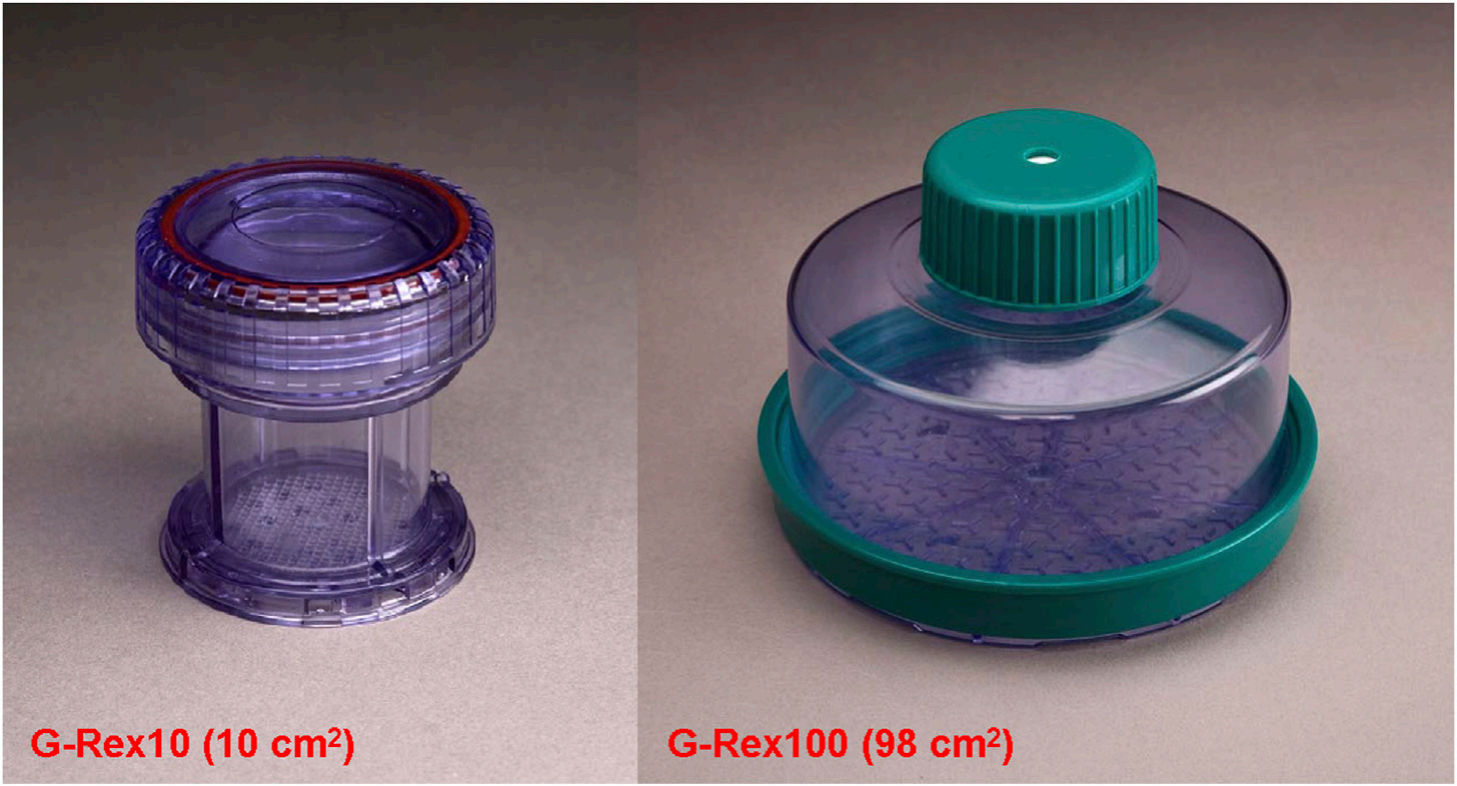
Two configurations of culture devices with gas-permeable (GP) silicone rubber membrane (SRM) bases; these are commercially available (Wilson Wolf Manufacturing). The G-Rex10 has a 10 cm^2^ area and is appropriate for research studies with up to 40,000 IE per device. The G-Rex100 has a 98 cm^2^ culture area and is suitable for the culture of entire human or porcine islet preparations, up to 400,000 IE per device. Versions of these models specially modified so that gas is displaced to avoid bubble formation can be used for the shipment of research or clinical islets.

The G-Rex10 and G-Rex100 devices (Figure 1) with areas of 2.5, 5, 10, 18, and 98 cm^2^ were used (Wilson Wolf Manufacturing, New Brighton, MN). The SRM bases of these devices have thicknesses between 152 and 229 μm (0.006–0.009 in); these allow the transfer of oxygen with very small diffusional resistance. Although larger thicknesses, up to 280 μm (0.011 in), were reported in some batches, modeling has shown that these do not limit oxygenation for ≤4,000 IE/cm^2^ over the observed range of islet oxygen consumption rate (OCR/DNA) (Papas et al., 2005; Kitzmann et al., 2014a).

### 2.5 Islet studies after 2 days of culture

#### 2.5.1 Measuring surface density by DNA content

Islet surface densities were measured by DNA content rather than by traditional manual counts of islets. Samples for DNA content were acquired at the initiation of the culture (T0) and at the end of the 2-day culture interval (T2). Samples for T0 were obtained by one of three methodologies (Shapiro et al., 2000): In most instances, devices were seeded based on calculations from manual IE counts but a sample for DNA content was taken from the pooled islet suspension (Hering et al., 2005); occasionally, cultures were seeded based solely on DNA content measured in the pooled islet suspension (Shapiro et al., 2006); in a few preparations, DNA samples were taken directly from the seeded devices at T0 to validate the accuracy of islet allocation and consequent surface density. DNA was determined using the Quant-iT Picogreen dsDNA kit (Molecular Probes, Eugene, OR).

#### 2.5.2 Calculating “DNA IE”

To express seeding cell densities in the more familiar nomenclature of islet equivalents (IE), DNA content was converted to DNA IE, in which 1.00 IE is defined as 10.4 ng DNA, the approximate DNA content of 1 IE (Colton et al., 2007). Values of “DNA IE” were applied to the islet cultures; however, quantitative DNA content in samples obtained directly from the OCR chamber was used in the calculation of OCR/DNA.

#### 2.5.3 Islet sampling

The islets were sampled randomly by pipetting to ensure that the aliquots were representative of each culture.

#### 2.5.4 Islet purity

Islet purity was visually assessed after dithizone staining in increments of 5% in two samples of at least 100 islets each (Ricordi, 1991; Papas et al., 2009). Purity was consistently 90%–95% for all preparations.

#### 2.5.5 Islet fractional viability assessment by OCR/DNA

Details of the OCR/DNA assay have been published previously (Papas et al., 2007a; Papas et al., 2007b; Colton et al., 2007). Briefly, several samples of islets (typically three samples, 1,000–2000 IE per sample) were placed in culture media and sealed inside a stainless steel chamber equipped with a fiberoptic sensor (Instech Laboratories, Plymouth Meeting, PA) that measures the partial pressure of oxygen (PO2) in the constantly stirred medium. The oxygen consumption rate (OCR) of each sample was calculated based upon the differential of PO2 measurements over the given time interval. The islets were then removed from the chamber by a series of washes and the concentration of DNA in the liquid was measured. From that, the total DNA in the chamber was calculated. To normalize the measured OCR to the number of cells in the chamber, the OCR is divided by the DNA content to yield the OCR/DNA value for that sample. The DNA assay has proven to be more sensitive than the fluorescein diacetate/propidium iodide (FDA/PI) cell membrane integrity assay which measures the upper bound of tissue viability (London et al., 1989) and therefore is less informative (Barnett et al., 2004; Papas et al., 2007a; Boyd et al., 2008; Kitzmann et al., 2014b; Papas et al., 2015a).

### 2.6 Diabetic nude mouse bioassay

Athymic nude mice were rendered diabetic with streptozotocin; diabetes was defined as a blood glucose level ≥350 mg/dL for at least 2 days pretransplant. Manually counted islets (2,000 IE) from the “standard” and “high-density GP” cultures were transplanted under the kidney capsule. Intact islets from the high-density *non-*GP condition were few in number (Supplementary Figure S1), rendering manual counts problematic. Instead, following DNA measurements post-culture, the DNA content was calculated, and the corresponding volume of 2,000 DNA IE transplanted. Diabetes reversal (DR) was defined as persistent blood glucose measurements ≤200 mg/dL (before nephrectomy on day 30 posttransplant). By convention, DR intervals are calculated from the day of transplant to the first day of the period of sustained normoglycemia.

### 2.7 Statistical analysis

Data are presented as arithmetic mean ± standard deviation (SD). The paired 2-sided Student’s *t-*test was used to assess statistical significance relative to the paired controls for non-categorical variables. Categorical comparisons between groups were performed using the 2-sided Fisher’s exact test (Agresti, 1992); comparison of DR time profiles was with the logrank test (Mantel, 1966). Significance was assessed at the *p* = 0.05 level for all tests. GraphPad Prism software (GraphPad Software Inc., La Jolla, CA) was used for all statistical calculations.

## 3 Results

### 3.1 Modeling of oxygen profiles

An important initial step in the design of the G-Rex flask configuration was to model high-density islet cultures seeded onto a silicon gas-permeable membrane versus a *non-*permeable membrane; both conditions were compared to the current standard: low-density cultures in T-flasks.

#### 3.1.1 Simulations of three different culture conditions

**FIGURE 2:**
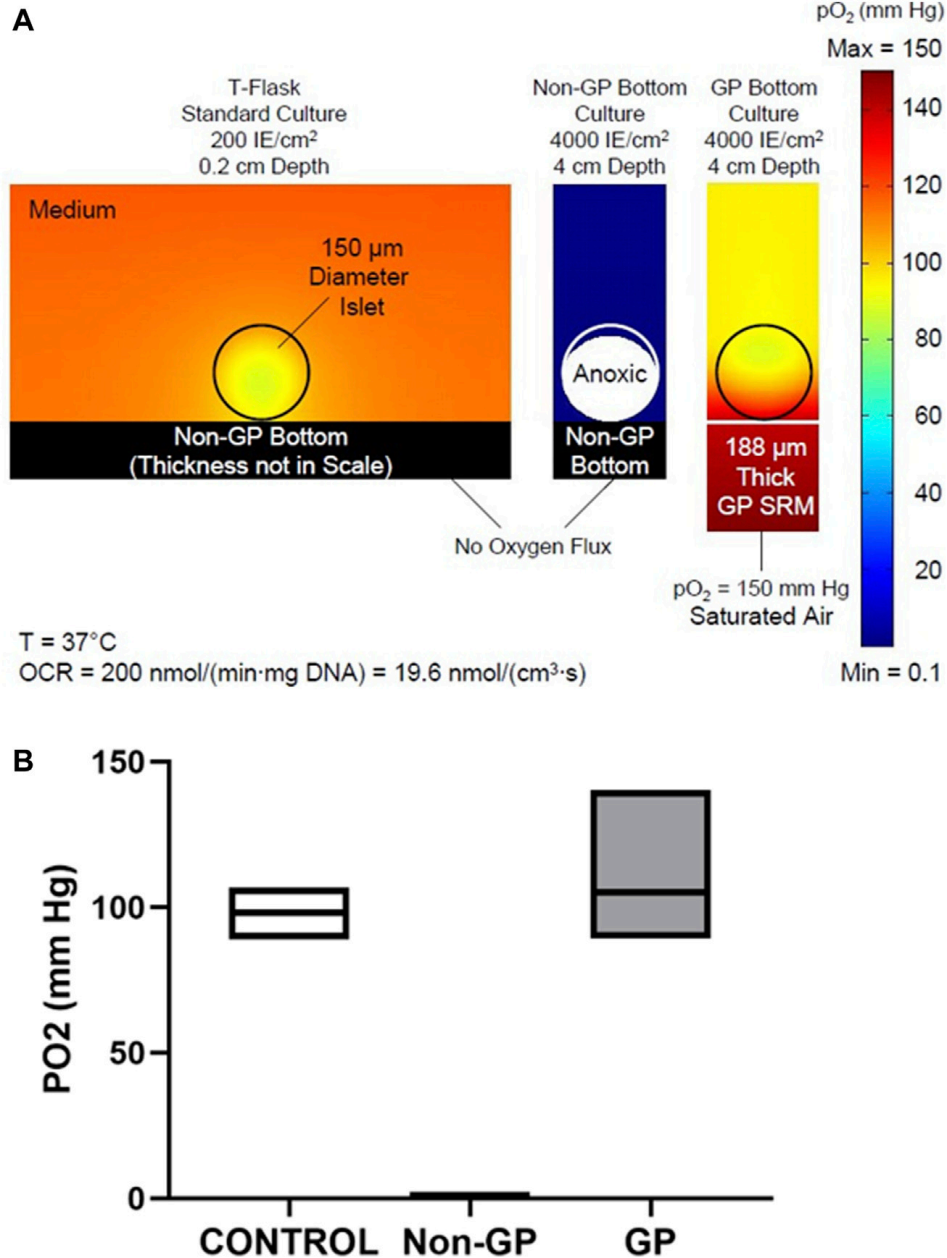
**(A)** Predicted oxygen partial pressure (PO2) surface plots in and around islets under three culture conditions: (Left) low-density standard culture condition; (Middle) high density in a *non-*gas-permeable (*non*-GP) device; or (Right) at high density in a GP device. Islets cultured under standard conditions or at high density in GP devices are fully oxygenated; however, islets cultured at high density in *non*-GP devices are almost completely anoxic. **(B)** Box plots of the predicted mean, minimum, and maximum PO2 values *inside* islets cultured under the three conditions described in **(A)**.

The finite element simulations of oxygen profiles under the three culture conditions–standard T-Flask conditions, high density cultures in *non-*GP flasks, and high density cultures in GP flasks–are shown in Figure 2. For the modeling, the oxygen consumption rate (OCR) was assigned a value of 200 nmol/min/mg DNA and the islets a diameter of 150 μm; it was also assumed that the islets were homogeneously dispersed on the bottom of the culture device. The surface plots of oxygen partial pressure (PO2) are displayed in Figure 2A. Islets cultured under standard conditions (left panel) and at a surface density of 4,000 IE/cm^2^ on the GP membrane (right panel) are predicted to be fully oxygenated; the minimum (“solid diamond”) was located closer to the center of the islet (13 μm below its center) in the control condition but at the apex of the islet in the 4,000 IE/cm^2^ GP simulation (Figure 2A). Islets cultured at 4,000 IE/cm^2^ in *non*-GP devices, however, are severely hypoxic (center panel): 95.3% of the culture volume is predicted to be anoxic (PO2 ≤ 0.1 mm Hg, the PO2 at which viability ceases). Figure 2B displays the minimum, maximum, and mean PO2 values from islet simulations under the same three conditions. The values for minimum PO2 are very similar under the standard condition, 88.9 mm Hg, and the 4,000 IE/cm^2^ GP culture condition, 89.3 mm Hg (Figure 2B). However, both the volume average and the maximum PO2 were higher in the 4,000 IE/cm^2^ GP condition relative to the control. In stark contrast, the maximum PO2 was predicted to be always less than 0.2 mm Hg under the 4,000 IE/cm^2^, *non-*GP condition (Figure 2B).

#### 3.1.2 Simulations of different OCRs

**FIGURE 3:**
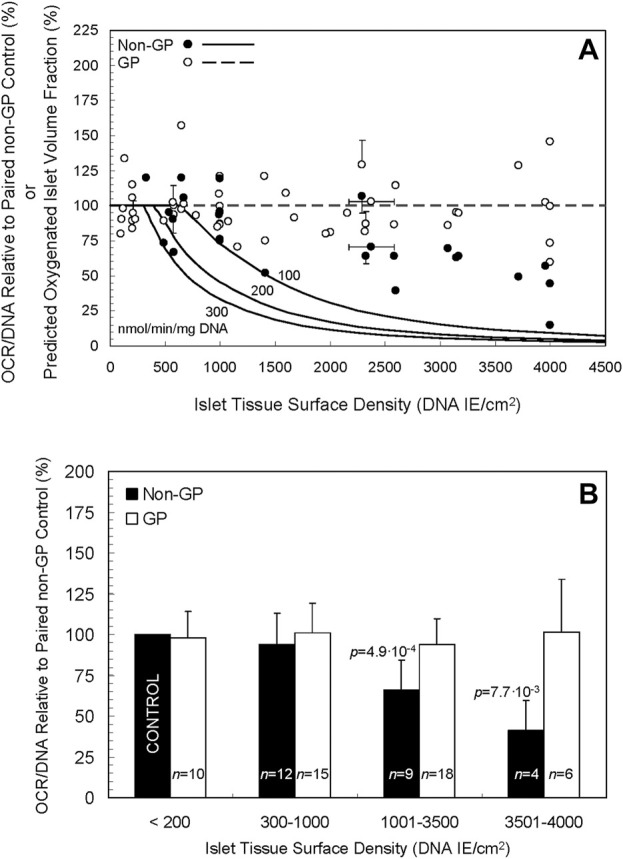
**(A)** Curves of three different oxygen consumption rates, 100, 200, and 300 nmol/min/mg DNA (solid lines), portray the *predicted* fraction of oxygenated islets by volume when cultured in *non*-GP flasks at a spectrum of islet tissue surface densities. The curves predict a rapid decline in the oxygenated fraction at surface densities > ∼ 500–600 DNA IE/cm^2^, especially at the higher two OCRs. Notably, the model predicts a consistent, fully oxygenated fractional volume (100%) in GP vessels (dashed line) at densities up to 4,500 DNA IE/cm^2^ and likely beyond. Also depicted are experimental data for OCR/DNA relative to paired controls for *non*-GP (filled circles) and GP (open circles) devices. Note that no data for *non*-GP devices are plotted below 200 DNA IE/cm^2^ as they are equivalent to controls that, after normalization, are by definition equal to 100%. The horizontal error bars denote the SD calculated by propagation of error from the coefficients of variation in the day 0 IE counts and DNA measurements from the islet pool; they do not incorporate errors associated with inaccurate seeding of the devices, a phenomenon that was verified for a small number of preparations by comparing the intended surface density (transformed to DNA) to that calculated from DNA samples taken on day 0 from the seeded devices. The vertical error bars denote the SD of the ratio calculated by propagation of error from the coefficient of variation associated with the values measured in each OCR chamber for the condition and its paired control. **(B)** The experimental data shown in A are plotted after combining the islet surface densities into four arbitrarily defined ranges. There is a statistically significant (*p* < 0.05) decrease in OCR/DNA relative to control for the 1,001–3,500 and 3,501–4,000 DNA IE/cm^2^ ranges for *non*-GP cultures.

The oxygenated volume fractions under GP and *non*-GP culture conditions were simulated under three OCR/DNA assumptions: 100, 200, and 300 nmol/min/mg DNA; these values represent typical low, moderate and high OCR/DNA values actually measured in porcine islet isolations. The simulations are portrayed *as curves* in Figure 3A (the same curves are reproduced in Supplementary Figures S2, S3). Even at the highest OCR/DNA value (300 nmol/min/mg DNA), and the highest surface density simulated (4,500 IE/cm^2^), 100% of the islet volume is predicted to be oxygenated in GP devices (Figure 3A, broken line). In sharp contrast, the first signs of central anoxia in *non-*GP devices were predicted at ∼310, ∼400, and ∼610 IE/cm^2^ for the three OCR/DNA values in descending order. No anoxia was predicted under control conditions (≤200 IE/cm^2^); therefore, the *predicted* oxygenated islet volume fractions shown in Figure 2A are unaffected by normalization to the control culture condition. Thus, the model predicts that a GP silicon membrane device would maintain the oxygenated volume fraction at 100% even at high (∼4,500 IE/cm^2^) islet density.

### 3.2 *In vitro* evaluation of cultured porcine islets in G-Rex devices

Two-day porcine islet cultures in prototype flasks with silicon membrane bases were evaluated for islet integrity and morphology, fractional viability, and functionality.

#### 3.2.1 Islet integrity and morphology

Purity was consistently 90%–95% for all preparations. Photomicrographs of islets following 2 days of culture documented the integrity and consistent morphology of islets cultured under either *standard conditions* (low surface density, *non*-GP) or at *high surface density in GP devices*. In sharp contrast, most islets cultured at *high surface density in non-GP devices* had disintegrated into single cells, Supplementary Figure S1. Manual IE counts for this condition were exceedingly low; therefore, to calculate islet mass (e.g., for the mouse bioassay) DNA content was employed (Materials and Methods, 2.4.4.).

#### 3.2.2 Sample islet viability by OCR and fractional viability assessment by OCR/DNA measurements

The OCR measurement is proportional to the total amount of viable tissue in the sample; DNA content is a measure of the total amount of tissue, both viable and nonviable. Therefore, the OCR divided by the DNA content of the sample (OCR/DNA ratio) is a representation of the fractional viability of the culture (Papas et al., 2007a; Papas et al., 2010; Suszynski et al., 2011; Kitzmann et al., 2014a).

##### 3.2.2.1 Post-culture OCR/DNA under control (standard) conditions

Mean OCR/DNA for the standard culture condition was 193.4 (n = 30, SD 45.1) nmol/min/mg DNA, a value which compares favorably with the 125–150 nmol/min/mg DNA threshold for diabetes reversal (DR) in diabetic nude mice receiving either human (Suszynski et al., 2011) or rat (Loganathan et al., 2010) islets; and the 175 nmol/min/mg DNA threshold required of porcine preparations for transplantation into non-human primates at our center (44, 45).

##### 3.2.2.2 Post-culture OCR/DNA of paired samples cultured in either GP or *non*-GP devices

The individual experimental OCR/DNAs are presented as scatter plots in Figure 3A, expressed relative to the OCR/DNA value of each condition’s paired control (a sample from the same islet preparation cultured under standard conditions). [The experimental data for the *non*-GP controls (filled circles) for surface seeding densities ≤200 IE/cm2 are by definition 100% and are not plotted.] For higher surface densities (>1,400 DNA IE/cm2) under *non*-GP conditions (filled circles), the fractional viability decreased significantly with increasing surface density but not to the extent predicted by the model (Figure 3A). For purposes of comparison with the model, the mean measured OCR/DNA on day 0 was 158 (n = 23, SD 42) nmol/min/mg DNA. For GP conditions (open circles), OCR/DNA values were largely unaffected by the surface density, a finding in agreement with the model. Coefficient of variation (COV) for OCR/DNA measurements from several OCR chambers is typically in the 10%–15% range, so the collective COV from two measurements is 15%–20% (10%–15% x √2).

The data shown in Figure 3A are replotted in Figure 3B after combining the normalized data into four groups, defined by arbitrary ranges of islet surface density. There was a statistically significant decrease in OCR/DNA relative to paired controls for the *non-*GP conditions in the 1,001–3,500 DNA IE/cm2 range (mean = 66, n = 9, SD 18, *p* = 4.9∙10-4), the 3,501–4,000 DNA IE/cm2 range (mean = 41, n = 4, SD 18, *p* = 7.7∙10-3), and the combined 1,001–4,000 DNA IE/cm2 group (mean = 58, n = 13, SD 21, *p* = 1.2∙10-5). Under GP conditions, however, OCR/DNA was largely unaffected by increasing surface density of islets (mean = 96, n = 24, SD 21, *p* = 0.33) relative to paired controls for the combined 1,001–4,000 DNA IE/cm2 range (Figure 3B). Together, the data indicate that the fractional viability of cultures on *non-*GP-membranes declined with increasing islet density, whereas the OCR/DNAs of GP cultures were consistently maintained at ∼100% throughout the range of islet density tested.

##### 3.2.2.3 Post-culture DNA content

Increases in OCR/DNA might be the consequence of increased OCR, decreased DNA content, or both. Therefore, we separately analyzed total (nonviable plus viable) DNA content, in comparison to the intentional DNA on day 0. These measurements showed significant deviations from the intended DNA surface density, likely due to islet sampling and allocation errors: average day 2 post-culture DNA recovery for the control condition was = 69.9% (n = 13, SD 22.4, *p* = 4.0∙10-4) relative to the day 0 value. Measured post-culture DNA recovery normalized to the DNA recovery of each condition’s paired controls are shown in Supplementary Figure S2A. For higher surface densities under *non*-GP conditions, DNA recovery was invariably lower than control values; for GP conditions, DNA recovery was always greater than controls. When grouped arbitrarily by the same surface densities as in Figure 3, there was a statistically significant decrease in DNA recovery relative to paired controls for the *non*-GP conditions in the 1,001–3,500, 3,501–4,000, and 1,001–4000 DNA IE/cm2 ranges. There was also a statistically significant increase in DNA recovery relative to paired controls for the GP condition in the ≤200, 1,001–3,500, 3,501–4,000, and the combined 1,001–4,000 DNA IE/cm2 ranges (Supplementary Figure S2B). These data indicate that DNA recovery followed the same trends as OCR/DNA but with greater increases under the GP condition.

##### 3.2.2.4 Post-culture OCR

Average post-culture OCR recovery (a measure of total active cellular metabolism independent of cell number) for the control condition was 97.5 (n = 11, SD 41.5, *p* = 0.85) relative to 100%. The measured post-culture OCR recovery relative to each condition’s paired controls is shown in Supplementary Figures S3A, B. Not surprisingly, the trends for total OCR mirror those for OCR/DNA but with wider excursions in the OCR values not normalized to DNA content.

In sum, OCR recovery, DNA recovery and OCR/DNA are increased in the GP cultures compared to the non-GP condition, especially at greater culture densities.

### 3.3 *In vivo* diabetes reversal (DR) by 2-day cultured islets

**FIGURE 4:**
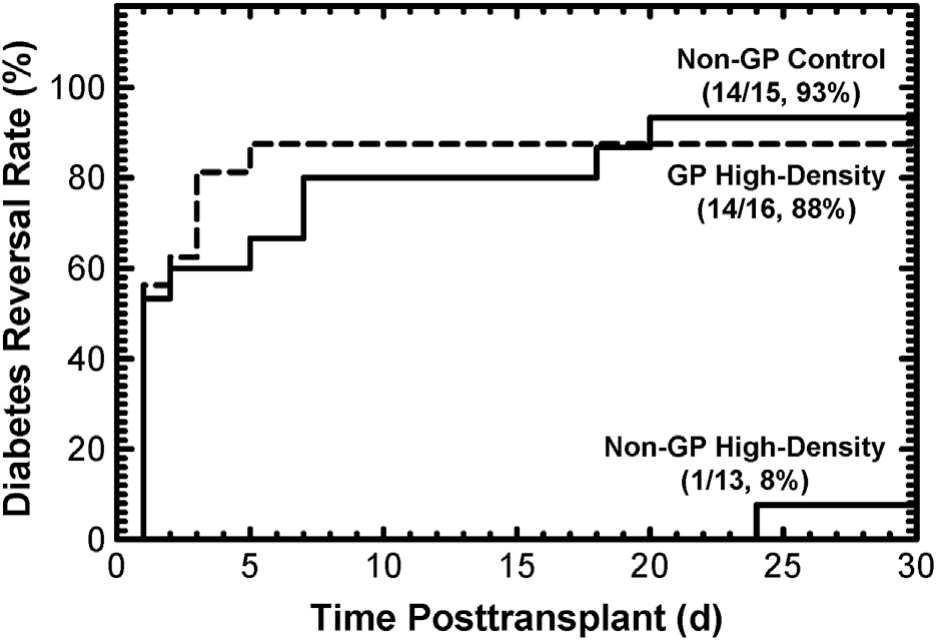
Diabetes reversal (DR) rates as a function of time posttransplant for diabetic athymic nude mice transplanted with 2,000 IE under the kidney capsule. Islets from 5 different preparations were used. Culturing under *non*-GP, high-density conditions resulted in destruction of the islets (Supplementary Figure S1), making transplantation based on manual IE counts impossible. Instead, the transplant “islet” doses were calculated based on the DNA content of 2,000 IE. The DR rates at the end of the 30-day posttransplant period were high (93% and 88%) for the control and the GP high-density conditions. In contrast, only 1 out of 13 mice transplanted with islet tissue cultured under *non*-GP high-density conditions resulted in (delayed) DR.

Both *in silico* modeling (Results, Section 3.1) and *in vitro* data (Results, Section 3.2) support the hypothesis that islets cultured at high densities in GP G-Rex devices meet or exceed the quality of conventional, low-density islet cultures. This supposition was tested *in vivo* using the diabetic athymic nude mice model, in which the reversal of diabetes by islets implanted under the kidney capsule was assessed. Islets from 5 preparations were cultured at surface densities ranging from 1,000 to 3,071 DNA IE/cm2, then implanted in two or three mice for each of the three conditions (Materials and Methods, 2.5). Because islets from the high-density*, non*-GP condition largely disintegrated during culture rendering manual counts of IE problematic, these mice were transplanted with an islet DNA content equal to that of 2,000 IE, to best approximate the paired controls. In a Kaplan-Meier plot, the DR rates (Figure 4) were similar for islets cultured at high densities (between 1,000 IE/cm2 and 3,071 DNA IE/cm2) in GP devices (14/16, 88%) as their *non-*GP control islets (14/15, 93%). In sharp contrast, only one of 13 mice (8%) transplanted with islets cultured at high densities in *non*-GP devices had reversal of its diabetes (*p* = 8.0∙10-6 relative to control, *p* = 2.2∙10-5 relative to high-density GP conditions); even the sole reversal was much-delayed. Application of the log rank test confirmed statistical significance (*p* < 1∙10-4) between the high-density *non*-GP and GP conditions.

## 4 Discussion

Currently, large-scale manufacture of islets requires islet culture; after isolating islets from a single pancreas, the conventional culture seeding density (≤200 IE/cm^2^) dictates large numbers of T-flasks and the attendant costs in labor, medium, incubator space and disposables. Our modeling of the oxygenated islet volume fraction (see Figure 3A) offers an explanation: at surface densities of ∼300–600 IE/cm^2^ (and depending on the OCR of the preparation), anoxia develops in the centers of 150 μm diameter islets. Uneven spatial distribution in the culture, as well as the presence of larger islets, further complicates oxygenation even at lower surface densities, hence the practical ≤200 IE/cm^2^ limit dictated by consensus and reinforced by experience.

There are four general approaches that have been explored to increase oxygen supply relative to oxygen demand in order to allow culturing islets at higher surface densities: a) *increase ambient PO2* above atmospheric levels; b) *decrease oxygen demand* by lowering culture temperature; c) *increase oxygen permeability* of the layer between the ambient oxygen and the islets; and d) *decrease the distance* between ambient oxygen and the islet layer.

Increased ambient PO2 has been tested with some success but is cumbersome and may cause hyperoxic damage. Decreasing oxygen demand by culturing at 22° failed to improve islet viability and recovery or lessen immunogenicity (Mueller et al., 2013), likely because the OCR at 22°C remains at about 30% of that at 37°C (Avgoustiniatos et al., 2008). Assuming a similar reduction in islet metabolic activity and a 3-4 fold increase in the islet volume density, and thus a 3-4 fold decrease in the medium depth, modeling predicts that islets with average OCR would experience anoxia at surface densities at or even below 2,000 IE/cm^2^. Even ignoring limitations in glucose diffusion, depletion of other nutrients, and accumulation of waste products, the established islet volume density of 1,000 IE/mL for 2-day culture could potentially be increased by a reduction in temperature, but only by a factor not more than two. Use of perfluorocarbons beneath the islet layer has been proposed for enhancing oxygenation during culture (Fraker et al., 2007). However, a perfluorocarbon layer sufficient to provide oxygen for 4000 IE/cm^2^ for 2 days of culture would have a depth of about 10 cm; that thickness would itself slow oxygen diffusion to the islets. The GP-SRM offers a remedy superior to these various approaches by *locating the ambient oxygen source immediately adjacent to the islet layer,* separated only by a gas-permeable membrane.

To increase the precision of OCR measurements and islet surface density and recovery assays, we chose to use DNA content as a measure of islet mass. The standard IE is a unit of islet volume, equal to the “prototypic” 150 μm diameter spherical islet. Conventional methods of counting by dithizone staining and light microscopy have been shown to overestimate the total number of IE by as much as 90% (Pisania et al., 2010). Although automated methods for islet counting limit operator variability (Friberg et al., 2011), they rely upon similar assumptions about islet geometry. DNA content measurement is more objective, operator-independent, and accounts for the presence of non-spherical islets and islets less than 50 μm in diameter. However, it cannot discriminate between islet and non-islet DNA; the latter is a minor issue with preparations of very high purity (90%–95%), such as the ones used in this study. The concept of DNA IE provides a convenient, familiar frame of reference; if all assumptions are met, it is equivalent to manually counted IEs.

Comparison of the *predicted* oxygen profiles reveals that oxygenation at high surface densities (4000 DNA IE/cm^2^) in GP devices is even better than in standard culture (≤200 DNA IE/cm^2^), as shown in Figure 2A. Although the minimum PO2 in the islets is almost identical (∼89 mm Hg), the maximum PO2 is notably higher in GP devices (140 mm Hg) relative to standard culture (107 mm Hg) (Figure 2B). How can 2 islets of the same size that consume oxygen with the same OCR (Michaelis-Menten effects on OCR being minimal at such high pO_2_ range) exhibit a large difference in ΔP (maximum PO2 - minimum PO2)? Islets cultured on GP membranes at high surface densities can be modeled as a “slab of tissue”, which for all practical purposes is oxygenated only from the surface of the slab abutting the membrane; the contribution of oxygen transport through the medium is negligible. In contrast, islets in standard culture at low density receive oxygen through the medium from all directions, except through the *non-*GP bottom; thus, the scattered islets resemble spheres in a semi-infinite medium. Hence it is not surprising that the ΔP in standard culture (107 – 89 = 18 mm Hg) is about 1/3 of that in a high-density culture in GP devices (140 – 89 = 51 mm Hg). This is predicted by diffusion theory and the Thiele modulus (Thiele, 1939) applied to spherical and planar geometries, given that the thickness of the slab (an islet monolayer at 4,000 IE/cm^2^) is 70.7 μm, quite similar to the radius of a single IE (75 μm). The impact of geometrical differences is also reflected in the location of the minimum pO_2_, which is just 13 μm below the center of the islet in standard culture but at the apex of the islet in high-density GP culture (black diamonds, Figure 2A). These observations are similar to those made when comparing the differences between spherical and slab geometry in the bioartificial pancreas (Avgoustiniatos et al., 1997; Johnson et al., 2009).

Importantly, the prediction of better oxygenation under high-density GP culture relative to standard culture conditions are reflected in the quite satisfactory morphologies of islets cultured at high surface densities in GP devices; the similar OCR/DNA (Figure 3), a more sensitive measure of viability than FDA/PI (Thiele, 1939; London et al., 1989; Barnett et al., 2004); OCR recovery relative to the standard culture control (Supplementary Figure S3); and the higher than control DNA recovery Supplementary Figure S2 (Boyd et al., 2008; Kitzmann et al., 2014b; Papas et al., 2015b). Finally, the therapeutic potency of islets, measured by the DR rate in diabetic nude mice, was high (88%) after a 2-day high density GP culture, results very similar to controls (Figure 4).

The theoretical prediction of anoxia in more than 90% of the islet volume at 4,000 IE/cm^2^ in *non-*GP devices, even for the low OCR value of 100 nmol/min/mg DNA, is dramatically illustrated by the nearly complete disintegration of islets (Supplementary Figure S1) and the extremely low DR rate with only one (delayed) DR out of 13 mice (Figure 4). It is also reflected in the large and statistically significant reductions post-culture in OCR/DNA, DNA, and OCR observed at high densities in *non-*GP devices, as shown in Figure 3; Supplementary Figures S2, S3. However, all 3 indices did not fall as rapidly with increasing surface densities as the model had predicted. The effects of anoxia on DNA integrity and its quantitative detection are not well characterized and may be complicated by residual degrading endogenous (Rose et al., 2003) or exogenous (Balamurugan et al., 2005; Loganathan et al., 2010) enzymes and the time course of DNA disintegration. However, if anoxic tissue completely degraded after 2 days of culture, one would expect that the recovery of total OCR, which is a measure of the total viable tissue, would be identical to the predicted oxygenated islet volume fraction. Indeed, OCR recovery more closely resembles the predictions than the OCR/DNA, although still not congruent with the models (Supplementary Figure S3A compared to Figure 3A). There are a number of plausible explanations for this difference, including: a) anoxia is less severe than predicted under the static culture assumption due to convection in the culture media (a phenomenon associated with the availability of glucose, otherwise limited by diffusion, in the depths of cultures); b) delays in establishing steady-state profiles as islets consume oxygen dissolved in the culture medium; c) variable susceptibility of islet cells to anoxia, as suggested by viability measurements under completely anoxic conditions; d) sporadic cell survival enabled by utilizing anaerobic metabolism; e) oxygenated tissue ceasing to consume oxygen for reasons other than unavailability (as suggested by the loss of IE and DNA under standard culture conditions); the remaining islets then advantageously compete for oxygen, a transition not reflected in simulations based on seeded surface density; f) restructuring of the tissue, as shown in Supplementary Figure S1; however, this likely plays only a minor role in tissue oxygenation simulation because at high surface densities the tissue continues to resemble a slab, whether composed of islets, single cells, or a mixture of both. Regardless of the explanation(s), despite the higher than modeled viable tissue (OCR) recovery after high-density *non*-GP culture, the loss of islet potency evidenced by the minimal DR rate (Figure 4) graphically emphasizes the severe functional effects of hypoxia at PO2s up to 100-fold greater than the lethal level (Einstein et al., 2023).

Similar GP devices are already in use for research and clinical studies of cell types other than islets: they increase the expansion of proliferating antigen-specific cytotoxic T lymphocytes (Avgoustiniatos et al., 2007; Avgoustiniatos et al., 2008), tumor infiltrating lymphocytes for adoptive cell therapy (Brandhorst et al., 1999; Kirchhof et al., 2004; Smith et al., 2018), cytokine-induced killer cells (Ricordi, 1991; Colton et al., 2007; Papas et al., 2009; Kitzmann et al., 2014a), regulatory T-cells (Papas et al., 2007a), virus and tumor-specific T cells (Avgoustiniatos et al., 2007; Papas et al., 2007b), and similar cell cultures for adoptive cell therapies (London et al., 1989; Boyd et al., 2008). GP devices have also been used to ship human islet preparations at high density (4,000 IE/cm^2^) from islet processing centers to distant laboratories, for both research and clinical applications (Barnett et al., 2004).

## 5 Conclusion

Porcine islet preparations were cultured for 2 days at surface densities as high as 4,000 DNA IE/cm^2^ in devices with gas-permeable, silicon rubber membrane bottoms without loss of islet integrity, viability (OCR/DNA), total tissue (DNA) recovery, total viable tissue (OCR) recovery, or islet potency (diabetes reversal rate in nude mice) relative to paired, standard culture controls. This constitutes a 20-fold increase in permissible islet surface density and a corresponding decrease in the number of devices required, making possible the culture of entire human or porcine islet preparations of high purity in one or two devices.

## Data Availability

The original contributions presented in the study are included in the article/Supplementary Material, further inquiries can be directed to the corresponding author.

## Abbreviations

COV, coefficient of variation; DNA IE, islet equivalent based on DNA content; DR, diabetes reversal; GP, gas permeable; HD, high density; IE, islet equivalent; *non-*GP, non-gas permeable; OCR, oxygen consumption rate; OCR/DNA:OCR normalized to DNA content, thus a measure of fractional viability; PO2, partial pressure of oxygen; SRM, silicon rubber membrane.

## References

[B1] Agresti A. (1992). A survey of exact inference for contingency tables. Stat. Sci. 7 (1), 131–177. 10.1214/ss/1177011454

[B2] Avgoustiniatos E. S. Colton C. K. (1997). “Design considerations in immunoisolation,” in Principles of tissue engineering. Editors Lanza R. P. Langer R. Chick W. L. (Austin, TX: RG Landes), 333–346.

[B3] Avgoustiniatos E. S. Colton C. K. (1997). Effect of external oxygen mass transfer resistances on viability of immunoisolated tissue. Ann. N. Y. Acad. Sci. 831, 145–167. 10.1111/j.1749-6632.1997.tb52192.x 9616709

[B4] Avgoustiniatos E. S. Dionne K. E. Wilson D. F. Yarmush M. L. Colton C. K. (2007). Measurements of the effective diffusion coefficient of oxygen in pancreatic islets. Ind. Eng. Chem. Res. 46(19):6157–6163. 10.1021/Ie070662y

[B5] Avgoustiniatos E. S. Hering B. J. Rozak P. R. Wilson J. R. Tempelman L. A. Balamurugan A. N. (2008). Commercially available gas-permeable cell culture bags may not prevent anoxia in cultured or shipped islets. Transpl. Proc. 40 (2), 395–400. 10.1016/j.transproceed.2008.01.059 PMC2764539 18374080

[B6] Balamurugan A. N. He J. Guo F. Stolz D. B. Bertera S. Geng X. (2005). Harmful delayed effects of exogenous isolation enzymes on isolated human islets: relevance to clinical transplantation. Am. J. Transpl. 5 (11), 2671–2681. 10.1111/j.1600-6143.2005.01078.x 16212626

[B7] Barnett M. J. McGhee-Wilson D. Shapiro A. M. Lakey J. R. (2004). Variation in human islet viability based on different membrane integrity stains. Cell. Transpl. 13 (5), 481–488. 10.3727/000000004783983701 15565860

[B8] Bellin M. D. Kandaswamy R. Parkey J. Zhang H. J. Liu B. Ihm S. H. (2008). Prolonged insulin independence after islet allotransplants in recipients with type 1 diabetes. Am. J. Transpl. 8 (11), 2463–2470. Epub 20080919. 10.1111/j.1600-6143.2008.02404.x PMC4312281 18808408

[B9] Boyd V. Cholewa O. M. Papas K. K. (2008). Limitations in the use of fluorescein diacetate/propidium iodide (Fda/Pi) and cell permeable nucleic acid stains for viability measurements of isolated islets of langerhans. Curr. Trends Biotechnol. Pharm. 2 (2), 66–84. 20814586 PMC2931281

[B10] Brandhorst H. Brandhorst D. Hering B. J. Bretzel R. G. (1999). Significant progress in porcine islet mass isolation utilizing liberase hi for enzymatic low-temperature pancreas digestion. Transplantation 68 (3), 355–361. 10.1097/00007890-199908150-00006 10459538

[B11] Colton C. K. Papas K. K. Pisania A. Rappel M. J. Powers D. E. O’Neil J. J. (2007). “Characterization of islet preparations,” in Cellular transplantation: from laboratory to clinic. Editors Halberstadt C. Emerich D. F. (Amsterdam ; Boston: Elsevier), 85–113.

[B12] Einstein S. A. Steyn L. V. Weegman B. P. Suszynski T. M. Sambanis A. O’Brien T. D. (2023). Hypoxia within subcutaneously implanted macroencapsulation devices limits the viability and functionality of densely loaded islets. Front. Transplant. 2, 2. 10.3389/frtra.2023.1257029 PMC11235299 38993891

[B13] Fiorina P. Shapiro A. M. Ricordi C. Secchi A. (2008). The clinical impact of islet transplantation. Am. J. Transpl. 8 (10), 1990–1997. 10.1111/j.1600-6143.2008.02353.x 18828765

[B14] Fraker C. A. Alvarez S. Papadopoulos P. Giraldo J. Gu W. Ricordi C. (2007). Enhanced oxygenation promotes β-cell differentiation *in vitro* . Stem Cells 25 (12), 3155–3164. 10.1634/stemcells.2007-0445 17761759

[B15] Friberg A. S. Brandhorst H. Buchwald P. Goto M. Ricordi C. Brandhorst D. (2011). Quantification of the islet product: presentation of a standardized current good manufacturing practices compliant system with minimal variability. Transplantation 91 (6), 677–683. Epub 2011/01/21. 10.1097/TP.0b013e31820ae48e 21248660

[B16] Hering B. J. Kandaswamy R. Ansite J. D. Eckman P. M. Nakano M. Sawada T. (2005). Single-donor, marginal-dose islet transplantation in patients with type 1 diabetes. JAMA 293 (7), 830–835. 10.1001/jama.293.7.830 15713772

[B17] Hogan A. Pileggi A. Ricordi C. (2008). Transplantation: current developments and future directions; the future of clinical islet transplantation as a cure for diabetes. Front. Biosci. 13, 1192–1205. 10.2741/2755 17981623

[B18] Johnson A. S. Fisher R. J. Weir G. C. Colton C. K. (2009). Oxygen consumption and diffusion in assemblages of respiring spheres: performance enhancement of a bioartificial pancreas. Chem. Eng. Sci. 64(22):4470–4487. 10.1016/j.ces.2009.06.028

[B19] Kirchhof N. Shibata S. Wijkstrom M. Kulick D. M. Salerno C. T. Clemmings S. M. (2004). Reversal of diabetes in non-immunosuppressed rhesus macaques by intraportal porcine islet xenografts precedes acute cellular rejection. Xenotransplantation 11 (5), 396–407. 10.1111/j.1399-3089.2004.00157.x 15303976

[B20] Kitzmann J. P. O’Gorman D. Kin T. Gruessner A. C. Senior P. Imes S. (2014b). Islet oxygen consumption rate dose predicts insulin independence for first clinical islet allotransplants. Transpl. Proc. 46 (6), 1985–1988. 10.1016/j.transproceed.2014.06.001 PMC4170186 25131089

[B21] Kitzmann J. P. Pepper A. R. Gala-Lopez B. Pawlick R. Kin T. O’Gorman D. (2014a). Human islet viability and function is maintained during high-density shipment in silicone rubber membrane vessels. Transpl. Proc. 46 (6), 1989–1991. 10.1016/j.transproceed.2014.06.002 PMC4169700 25131090

[B22] Loganathan G. Dawra R. K. Pugazhenthi S. Wiseman A. C. Sanders M. A. Saluja A. K. (2010). Culture of impure human islet fractions in the presence of alpha-1 antitrypsin prevents insulin cleavage and improves islet recovery. Transpl. Proc. 42 (6), 2055–2057. 10.1016/j.transproceed.2010.05.119 PMC2924667 20692406

[B23] London N. J. Contractor H. Lake S. P. Aucott G. C. Bell P. R. James R. F. (1989). A microfluorometric viability assay for isolated human and rat islets of langerhans. Diabetes Res. 12 (3), 141–149. Epub 1989/11/01. 2699586

[B24] Mantel N. (1966). Evaluation of survival data and two New rank order statistics arising in its consideration. Cancer Chemother. Rep. 50 (3), 163–170. 5910392

[B25] Mourad N. I. Gianello P. R. (2017). Xenoislets: porcine pancreatic islets for the treatment of type I diabetes. Curr. Opin. Organ Transpl. 22 (6), 529–534. 10.1097/mot.0000000000000464 28915137

[B26] Mueller K. R. Martins K. V. Murtaugh M. P. Schuurman H. J. Papas K. K. (2013). Manufacturing porcine islets: culture at 22 °C has no advantage above culture at 37 °C: a gene expression evaluation. Xenotransplantation 20 (6), 418–428. Epub 20130814. 10.1111/xen.12048 23941232 PMC3849224

[B27] Nason R. W. Rajotte R. V. Warnock G. L. (1988). Pancreatic islet cell transplantation: past, present and future. Diabetes Res. 7 (1), 1–11. 3135975

[B28] Papas K. K. Avgoustiniatos E. S. Tempelman L. A. Weir G. C. Colton C. K. Pisania A. (2005). High-density culture of human islets on top of silicone rubber membranes. Transpl. Proc. 37 (8), 3412–3414. 10.1016/j.transproceed.2005.09.086 16298611

[B29] Papas K. K. Bellin M. D. Sutherland D. E. Suszynski T. M. Kitzmann J. P. Avgoustiniatos E. S. (2015a). Islet oxygen consumption rate (ocr) dose predicts insulin independence in clinical islet autotransplantation. PLoS One 10 (8), e0134428. Epub 20150810. 10.1371/journal.pone.0134428 26258815 PMC4530873

[B30] Papas K. K. Bellin M. D. Sutherland D. E. Suszynski T. M. Kitzmann J. P. Avgoustiniatos E. S. (2015b). Islet oxygen consumption rate (ocr) dose predicts insulin independence in clinical islet autotransplantation. PLoS ONE 10 (8), e0134428. 10.1371/journal.pone.0134428 26258815 PMC4530873

[B31] Papas K. K. Colton C. K. Nelson R. A. Rozak P. R. Avgoustiniatos E. S. Scott W. E. 3rd (2007a). Human islet oxygen consumption rate and DNA measurements predict diabetes reversal in nude mice. Am. J. Transpl. 7 (3), 707–713. Epub 20070104. 10.1111/j.1600-6143.2006.01655.x PMC2857994 17229069

[B32] Papas K. K. Colton C. K. Qipo A. Wu H. Nelson R. A. Hering B. J. (2010). Prediction of marginal mass required for successful islet transplantation. J. Investig. Surg. 23 (1), 28–34. 10.3109/08941930903410825 20233002 PMC3786417

[B33] Papas K. K. Pisania A. Wu H. Weir G. C. Colton C. K. (2007b). A stirred microchamber for oxygen consumption rate measurements with pancreatic islets. Biotechnol. Bioeng. 98 (5), 1071–1082. Epub 2007/05/15. 10.1002/bit.21486 17497731 PMC2859188

[B34] Papas K. K. Suszynski T. M. Colton C. K. (2009). Islet assessment for transplantation. Curr. Opin. Organ Transpl. 14 (6), 674–682. 10.1097/MOT.0b013e328332a489 PMC2859186 19812494

[B35] Pisania A. Papas K. K. Powers D. E. Rappel M. J. Omer A. Bonner-Weir S. (2010). Enumeration of islets by nuclei counting and light microscopic analysis. Lab. Investig. 90 (11), 1676–1686. Epub 20100809. 10.1038/labinvest.2010.125 20697375 PMC2966546

[B36] Protocols C. I. T. (2015). Purified human pancreatic islets master production batch record. Available at: http://www.isletstudy.org/CITDocs/SOP%203101,%20B01,%20MPBR%20v05,%20October%2028,%202010.pdf:NIH October 28, 2010).

[B37] Ricordi C. (1991). Quantitative and qualitative standards for islet isolation assessment in humans and large mammals. Pancreas 6 (2), 242–244. 10.1097/00006676-199103000-00018 1679542

[B38] Rose N. L. Palcic M. M. Lakey J. R. (2003). An evaluation of endogenous pancreatic enzyme levels after human islet isolation. Pancreas 27 (2), 167–173. 10.1097/00006676-200308000-00010 12883266

[B39] Shapiro A. M. Lakey J. R. Ryan E. A. Korbutt G. S. Toth E. Warnock G. L. (2000). Islet transplantation in seven patients with type 1 diabetes mellitus using a glucocorticoid-free immunosuppressive regimen. N. Engl. J. Med. 343 (4), 230–238. Epub 2000/07/27. 10.1056/nejm200007273430401 10911004

[B40] Shapiro A. M. Ricordi C. Hering B. J. Auchincloss H. Lindblad R. Robertson R. P. (2006). International trial of the Edmonton protocol for islet transplantation. N. Engl. J. Med. 355 (13), 1318–1330. 10.1056/NEJMoa061267 17005949

[B41] Smith K. E. Purvis W. G. Davis M. A. Min C. G. Cooksey A. M. Weber C. S. (2018). *In vitro* characterization of neonatal, juvenile, and adult porcine islet oxygen demand, β‐cell function, and transcriptomes. Xenotransplantation 25 (6), e12432. 10.1111/xen.12432 30052287

[B42] Suszynski T. M. Avgoustiniatos E. S. Stein S. A. Falde E. J. Hammer B. E. Papas K. K. (2011). Assessment of tissue-engineered islet graft viability by fluorine magnetic resonance spectroscopy. Transpl. Proc. 43 (9), 3221–3225. 10.1016/j.transproceed.2011.09.009 PMC4757485 22099762

[B43] Thiele E. W. (1939). Relation between catalytic activity and size of particle. Industrial Eng. Chem. 31 (7), 916–920. 10.1021/ie50355a027

[B44] Yeager T. Roy S. (2017). Evolution of gas permeable membranes for extracorporeal membrane oxygenation. Artif. Organs 41 (8), 700–709. 10.1111/aor.12835 28105685

