# Supplementary material for: Silicone rubber membrane devices permit islet culture at high density without adverse effects
Source: Front Bioeng Biotechnol. 2024 Jul 11;12:1401608. doi: 10.3389/fbioe.2024.1401608 (PMC11273363; doi:10.3389/fbioe.2024.1401608)
Supplement: Supplementary file 1 [file DataSheet1.pdf]

Supplementary Material

Silicone Rubber Membrane Devices Permit Islet Culture at High Density without Adverse Effects[2]22

Efstathios S. Avgoustiniatos[1], Kate R. Mueller, William E. Scott III, Jennifer P. Kitzmann, Thomas M. Suszynski, Brian E. Perrault, Eric J. Falde, Phillip R. Rozak, A.N. Balamurugan, Bernhard J. Hering, Charles W. Putnam, Klearchos K. Papas*

[1]Correspondence regarding modeling: eavgoust@alum.mit.edu

***Correspondence:** Corresponding Author: Klearchos K. Papas kkpapas@surgery.arizona.edu

1 Modeling of oxygen profiles

2 Supplementary Figures

1 Modeling of oxygen profiles

We assumed spherical islets of uniform 150-μm diameter cultured in a square array configuration. A confluent islet monolayer under this configuration corresponds to a surface coverage of $\pi/4 \simeq 78.5\%$ or 4444 IE/cm^2. For standard culture the medium depth was varied to maintain a constant tissue-to-medium volume ratio of 1000 IE/mL (current standard). For example, for 4000 IE//cm^2 the medium depth used was 40 mm. For culture on SRM, the medium depth was kept constant at 50 mm regardless of surface coverage, as oxygenation through the medium was not crucial.

Equilibrium is established very fast through the SRM while oxygen delivery from the medium side is comparatively very small and so any potential time variation can be ignored. Therefore, this can be considered a pseudo-steady-state problem with no time variation.

The steady-state diffusion-reaction equation is

$$D\alpha\,\nabla^2 pO_2 = V$$

where D is the local oxygen diffusivity [cm^2/s], α is the local oxygen solubility [mol/(cm^3·mm Hg)] and V is the local volumetric oxygen consumption rate [mol/(cm^3·s)].

The use of partial pressures instead of concentrations provides the convenience of pO_2 continuity at every interface, negating the need for defining partition coefficients. For a steady-state or pseudo-steady-state problem like this, only the product Dα, known as oxygen permeability, is necessary in each phase.

It was assumed that islet tissue consumes oxygen with a uniform maximum volumetric rate Vmax following Michaelis-Menten kinetics with a Km value of ≃ 0.44 mm Hg, down to a critical pO_2 cut-off value of 0.1 mm Hg. This cut-off value was also used to calculate the reported oxygenated or anoxic volume fractions. For fully viable tissue a Michaelis-Menten Vmax of 19.6 nmol/(cm^3 · s), corresponding to 200 nmol/(min · mg DNA) was assumed. Hence the local oxygen consumption rate inside the islet is

$$V = \mathrm{Vmax}\,\frac{pO_2}{pO_2 + Km} \text{ for } pO_2 \geq 0.1 \text{ mm Hg}$$

while V = 0 everywhere else.

This diffusion-reaction problem is a 3-D one but it has been shown (1) that azimuthal variations can be ignored and the problem solved for all practical purposes as an axisymmetric 2-D problem by assuming the same tissue load per unit area.

We assumed a constant external pO_2 value of 150 mm Hg, corresponding to fully humidified air at 1 atm without additional CO_2. Negligible oxygen gradients in the gas phase were assumed. The silicone rubber membrane (SRM) thickness was assigned a value of 188 μm based on the average thickness of individual membranes from a single batch, mean 188 (SD 3) μm (1). The other parameters used in the modeling are described elsewhere (1-4).

With these assumptions, the diffusion-reaction equation was solved using the COMSOL Multiphysics finite-element simulation package (COMSOL Inc., Burlington, MA).

References

1. Avgoustiniatos ES, Hering BJ, Rozak PR, Wilson JR, Tempelman LA, Balamurugan AN, et al. Commercially Available Gas-Permeable Cell Culture Bags May Not Prevent Anoxia in Cultured or Shipped Islets. Transplant Proc (2008) 40(2):395-400. doi:https://doi.org/ 10.1016/j.transproceed.2008.01.059.

2. Avgoustiniatos ES, Colton CK. Effect of External Oxygen Mass Transfer Resistances on Viability of Immunoisolated Tissue. Ann N Y Acad Sci (1997) 831:145-67. doi:https://doi.org/ 10.1111/j.1749-6632.1997.tb52192.x.

3. Avgoustiniatos ES, Dionne KE, Wilson DF, Yarmush ML, Colton CK. Measurements of the Effective Diffusion Coefficient of Oxygen in Pancreatic Islets. Ind Eng Chem Res (2007) 46(19):6157-63. doi:https://doi.org/ Doi 10.1021/Ie070662y.

4. Papas KK, Avgoustiniatos ES, Tempelman LA, Weir GC, Colton CK, Pisania A, et al. High-Density Culture of Human Islets on Top of Silicone Rubber Membranes. Transplant Proc (2005) 37(8):3412-4. doi:https://doi.org/ 10.1016/j.transproceed.2005.09.086.

2 Figures

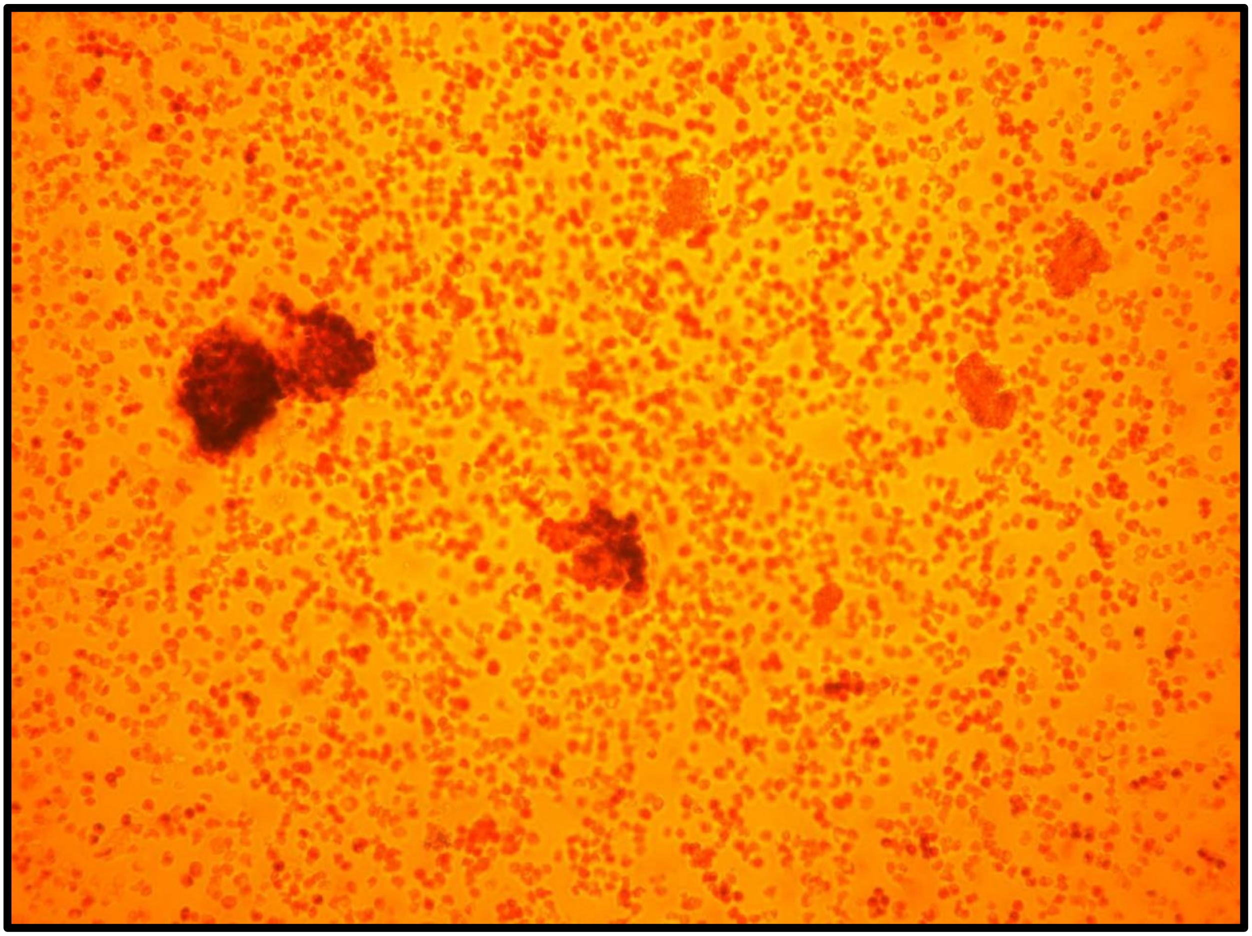

Supplementary Figure 1. Photomicrograph of dithizone-stained islets cultured for 2 days at a high surface density (4000 IE/cm^2 or 2300 DNA IE/cm^2) in a non-GP device. Nearly all islets have disintegrated into single cells.

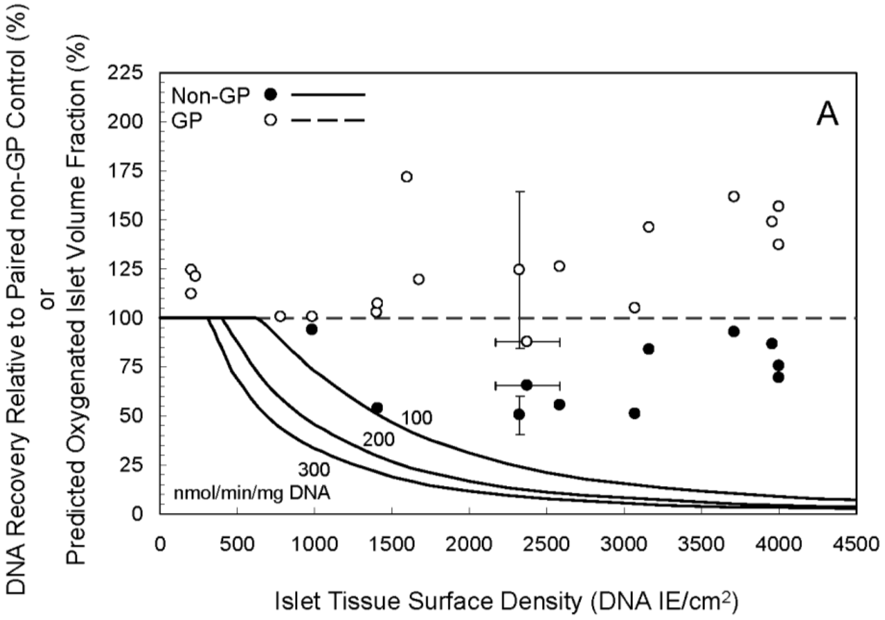

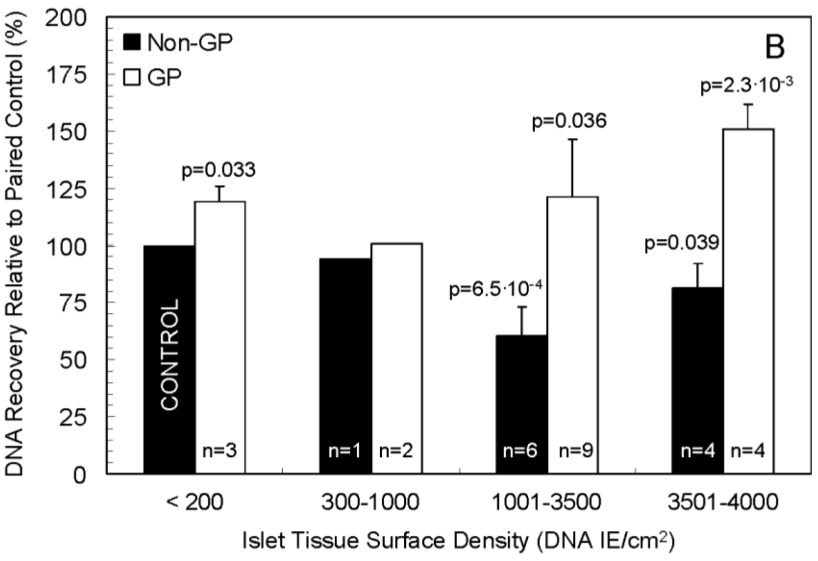

Supplementary Figure S2. A. Experimental data (circles) for DNA recovery relative to paired controls for non-gas-permeable (non-GP) and GP devices. The modeled lines are the same as in **Figure 3**. Both experimental data and the simulations show a decline with increasing surface (seeding) density under non-GP conditions but not under GP conditions. The vertical error bars denote the SD of the DNA recovery ratio calculated by propagation of error from the coefficients of variation associated with the day 0 and day 2 DNA measurements for both the condition and its paired control. COV for each DNA measurement is typically in the 10-15% range, so the collective COV from 4 measurements was 20-30% (10-15% x √4). B. The same experimental data plotted in A are shown after combining them into 4 arbitrarily defined islet surface density ranges. There is

a statistically significant decrease in DNA recovery relative to controls for the 1001-3500 and 3501-4000 DNA IE/cm^2 ranges for non-GP cultures and a statistically significant increase for 3 of the 4 ranges for GP cultures.

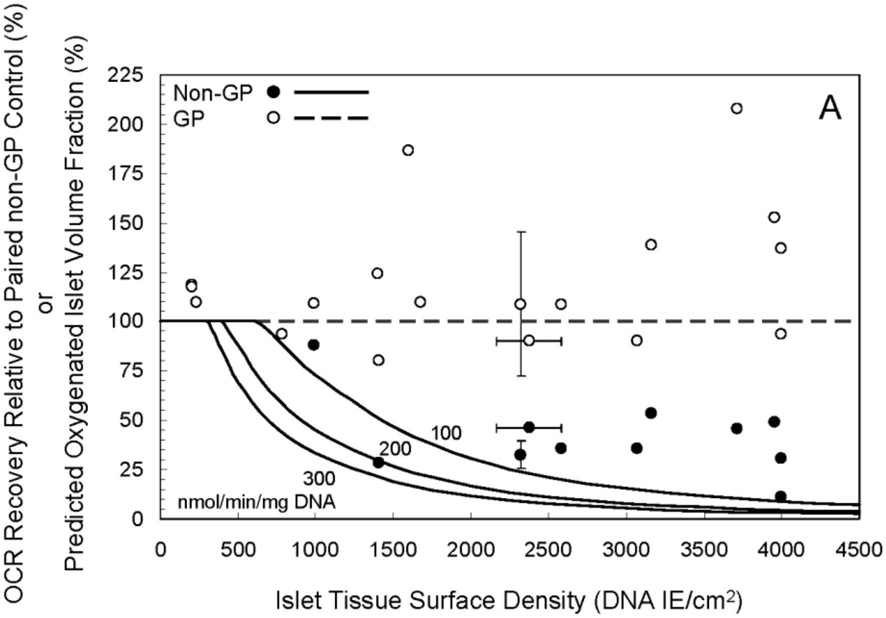

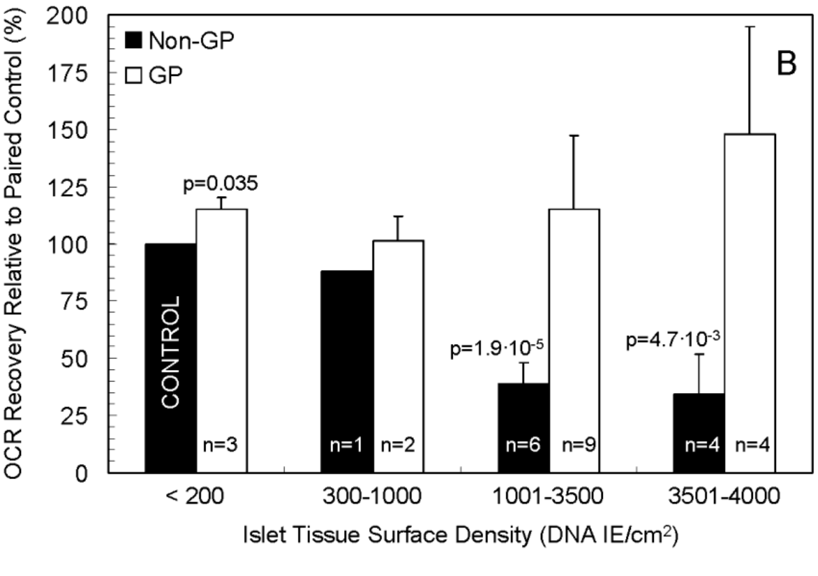

Supplementary Figure S3. A. Experimental data (circles) for OCR recovery relative to paired controls for non-GP and GP devices. The curves are the same as in **Figures 3** and **S2**. Both the simulation model and the experimental data show a decline with increasing surface density under non-GP conditions but not under GP conditions. The vertical error bars denote the SD of the OCR recovery ratio calculated by propagation of error from the coefficients of variation in OCR/DNA and DNA recovery, resulting in a collective COV of 25-35% (10-15% x $\sqrt{6}$). **B**. The same experimental data shown in **A** have been combined into four arbitrarily defined islet surface density ranges. There is a statistically significant decrease in DNA recovery relative to control for the 1001-3500 and 3501-4000 DNA IE/cm^2 range for non-GP cultures and a statistically significant increase for the < 200 DNA IE/cm^2 range for GP culture.
